# PLASMA BD‐TAU AS A SPECIFIC CORRELATE OF DISEASE SEVERITY AND DYNAMIC ACROSS THE ALZHEIMER'S DISEASE SPECTRUM

**DOI:** 10.1002/alz.088853

**Published:** 2025-01-09

**Authors:** Giuseppe Piga, Fernando Gonzalez‐Ortiz, Idil Yuksekel, Marion Houot, Laura Gonzalez Uribe, Armelle Rametti‐Lacroux, Marc Teichmann, Stéphanie Bombois, Isabelle Le Ber, Bruno Dubois, Richard Levy, Foudil Lamari, Kaj Blennow, Nicolas Villain

**Affiliations:** ^1^ Hôpital Pitié‐Salpêtrière, Département de Neurologie, Institut de la Mémoire et de la Maladie d'Alzheimer, Paris, Ile de France France; ^2^ University of Gothenburg, Mölndal Sweden; ^3^ Sorbonne Université, INSERM U1127, CNRS 7225, Paris Brain Institute ‐ ICM, Paris France; ^4^ Sorbonne Université, Paris Brain Institute (ICM), INSERM, CNRS, CENIR MEG‐EEG, Paris France; ^5^ Hôpital Pitié‐Salpêtrière, Département de Neurologie, Institut de la Mémoire et de la Maladie d'Alzheimer, Paris France; ^6^ Inserm U 1127, CNRS UMR 7225, Sorbonne Universités, UPMC Univ Paris 06, ICM, Paris France; ^7^ AP‐HP Sorbonne Université, Hôpital Pitié‐Salpêtrière, Department of Neurology, Paris France; ^8^ Assistance Publique Hopitaux de Paris (APHP), Paris France; ^9^ Département de Neurologie, AP‐HP‐Hôpital Pitié‐Salpêtrière, Paris France; ^10^ Sorbonne Université, Paris Brain Institute – Institut du Cerveau – ICM, Inserm U1127, CNRS UMR 7225, AP‐HP ‐ Hôpital Pitié‐Salpêtrière, Paris France; ^11^ Sorbonne Université, INSERM U1127, CNRS 7225, Paris France; ^12^ AP‐HP, Groupe Hospitalier Pitié‐Salpêtrière, Department of Neurology, IM2A, Paris France; ^13^ Service de Biochimie Métabolique, Hôpital Pitié‐Salpêtrière, Paris, France, Paris France; ^14^ Paris Brain Institute, ICM, Pitié‐Salpêtrière Hospital, Sorbonne University, Paris France; ^15^ Institute of Neuroscience and Physiology, Department of Psychiatry and Neurochemistry, The Sahlgrenska Academy, University of Gothenburg, Mölndal Sweden

## Abstract

**Background:**

The identification of biomarkers for Alzheimer's disease (AD) remains a significant challenge, particularly for the clinical severity of the disease. Recent studies have shown that plasma brain‐derived‐tau (BD‐Tau) could be a promising biomarker for the identification of AD‐type neurodegeneration. This study aimed to investigate the potential of BD‐Tau in differentiating various clinical stages of AD, ranging from cognitively unimpaired AD to severe dementia AD. Additionally, the study intended to examine the association of BD‐Tau with clinical severity and elucidate its dynamic behavior throughout the natural course of AD.

**Method:**

Cross‐sectional and longitudinal EDTA plasma data, along with clinical information, were utilized from the Pitié‐Salpêtrière hospital INSIGHT (cognitively unimpaired individuals with amyloid PET) and SOCRATES cohorts (AD and non‐AD symptomatic neurodegenerative disease according to up‐to‐date international criteria). Plasma BD‐Tau was analyzed using the Gothenburg University homebrew Quanterix Simoa immunoassay.

**Result:**

The results revealed that BD‐Tau is only elevated in individuals with symptomatic AD, whether the primary or secondary neurodegenerative disease, and not in other neurodegenerative conditions. Additionally, BD‐Tau follows the clinical dynamics of AD, as it is only elevated in symptomatic AD patients, while individuals with AD biological changes but no symptoms have normal levels of BD‐Tau. BD‐Tau levels were also found to be significantly correlated with episodic memory scores and global cognitive performance across a wide range of AD severity. Longitudinal analyses further support BD‐Tau's potential as an AD‐type neurodegeneration biomarker, as it was found to increase over time only in the symptomatic AD group and not in other groups.

**Conclusion:**

These results support the notion that plasma BD‐Tau could be a promising specific biomarker for AD staging, as it follows the AD clinical severity spectrum and is not elevated in asymptomatic amyloidosis.

